# IPUMS MULTIGENERATIONAL LONGITUDINAL PANEL

**DOI:** 10.1093/geroni/igae098.1752

**Published:** 2024-12-31

**Authors:** Catherine Fitch, Steven Ruggles

**Affiliations:** University of Minnesota, Minneapolis, Minnesota, United States; University of Minnesota, Minneapolis, Minnesota, United States

## Abstract

Over the past five years, the IPUMS Multigenerational Longitudinal Panel (MLP) has constructed a massive longitudinal population panel by linking together American censuses, surveys, administrative sources, and vital records spanning the period from 1850 to the present. MLP is designed as a general-purpose resource for studying longitudinal processes and long-run social and economic change. By linking individuals across generations from birth through death using data from multiple sources, MLP enables reproducible research on shifting patterns of life course patterns and intergenerational processes. The large scale of the MLP database allows researchers both to study particular communities and small dispersed populations and to conduct big studies spanning many places and periods. Mounting evidence shows that early life social, economic, policy, and environmental exposures are important drivers of later life cognitive, health, and economic outcomes. MLP creates unprecedented opportunities for prospective analyses of the impact of early-life exposures on a wide range of later-life outcomes.

